# PERMA-based PBL teaching model: a case study of mechanical drawing in vocational education

**DOI:** 10.3389/fpsyg.2025.1536891

**Published:** 2025-07-21

**Authors:** Yiming Lu, Yonghuan Guo, Xiwen Wang

**Affiliations:** School of Mechatronic Engineering, Jiangsu Normal University, Xuzhou, China

**Keywords:** PERMA model, PBL teaching model, positive psychology, vocational education, mechanical drawing

## Abstract

In mainland China, the mental health of vocational high school students has been increasingly recognized as a critical issue by both the government and scholars. Positive education, a branch of positive psychology, has been proven to significantly enhance adolescents’ well-being and academic performance. Exploring how to effectively integrate positive education theories or models with traditional teaching methods in vocational classrooms is a promising research direction. To address this, the present study uses the *Mechanical Drawing* course as a case study, combining the PERMA model with the traditional PBL teaching model to propose an innovative and practical teaching method: the PERMA-based PBL teaching model. The study involved 207 first-year vocational high school students from four parallel classes. Based on their pre-test scores, the students were divided into two parallel groups (A and B) for a crossover experimental design. Quantitative data were collected using the Inventory of Positive Mental Characters among Chinese Middle School Students and a course survey questionnaire. Each group underwent pre-test, mid-test, and post-test assessments, with quantitative analysis conducted via t-tests to evaluate the advantages of the new teaching model compared to traditional teaching methods. The results indicated that the new teaching model significantly outperformed the traditional teaching model in enhancing all dimensions of vocational high school students’ positive psychological qualities (*p* < 0.001). Additionally, the new teaching model was superior in improving students’ Learning Interest (*p* < 0.001), Learning Motivation (*p* < 0.001), and Learning Attitude (*p* < 0.05). Furthermore, vocational students expressed a stronger preference for the PERMA-based PBL teaching model compared to the traditional teaching model (*p* < 0.001). This study explores a practical pathway for integrating positive psychology theories into classroom settings and provides a reference for future research in this field.

## 1 Introduction

Currently, a significant number of adolescents are experiencing poor mental health. According to the World Health Organization (2022), approximately 14% of adolescents aged 10–19 globally suffer from mental disorders. Similarly, a study conducted in mainland China revealed that about 17.5% of adolescents have poor mental health levels (Li et al., 2022), with this issue being particularly severe among vocational high school students (He et al., 2023). In mainland China, vocational high school students generally perform worse academically in their middle school years compared to general high school students and tend to have lower cognitive motivation (Liu and Jiang, 2020). However, vocational students are required to master both academic content and additional work-related skills, which may result in differences in their mental health outcomes compared to general high school students (Su et al., 2024). Previous research has shown that vocational students exhibit lower psychological resilience compared to their general high school peers (Lu et al. 2025). Moreover, they may have a higher incidence of self-harming behaviors and are more likely to experience suicidal ideation (Horváth et al., 2018).

On the other hand, students’ mental health and academic performance are mutually reinforcing (Seligman, 2012). Poor mental health negatively impacts students’ academic self-efficacy, progress, persistence, and daily performance (Leow et al., 2024; Chim et al., 2024). Conversely, students with better mental health and higher levels of well-being tend to achieve better academic outcomes, possess more adaptive motivational beliefs, and establish more positive relationships with teachers, parents, and peers (King et al., 2023). Adolescents’ positive emotions are significantly correlated with enhanced creativity, prosocial behaviors, problem-solving and coping abilities, as well as positive perceptions of themselves and others (Lyubomirsky et al., 2005). Moreover, positive emotions and well-being serve as protective factors against adolescent depression and play a crucial role in promoting mental health (Zhao et al., 2019). Therefore, fostering positive emotions and well-being among vocational high school students is essential for improving both their academic performance and mental health.

A good trend is that positive psychology has gained increasing attention from educators across Asia in recent years (Lim, 2024). According to the Global Happiness Council, over 10,000 schools in mainland China have incorporated principles of positive psychology into their teaching practices (Global Happiness Council, 2018). In 2012, Seligman introduced the PERMA model, which identifies five core elements of well-being: Positive emotion, Engagement, Relationships, Meaning, and Accomplishment (Seligman, 2012). As a classic model within the discipline of positive psychology, the PERMA model has demonstrated significant effectiveness in enhancing adolescents’ psychological resilience (Falecki et al., 2018) and in mitigating negative emotions and mental health disorders (Kern et al., 2015). Consequently, the PERMA model has been widely adopted in schools to develop positive psychology interventions (Lan and Saad, 2024; Morgan and Simmons, 2021) and for psychological assessments (Lai et al., 2018; Chue et al., 2024). Its influence is reaching an increasing number of students.

However, implementing the PERMA model and the other model of positive psychology in vocational high schools in mainland China still faces numerous challenges. First, the number of dedicated school psychologic counselors in vocational schools remains insufficient. A study focusing on Sichuan Province revealed that only about 77% of vocational schools in the region are staffed with full-time school psychologic counselors, with the total number of such counselors accounting for just 0.049% of the total vocational student population. The professional competence of other instructors also exhibits uneven development (Dong et al., 2020), with limited understanding of the PERMA model and related theories in positive psychology. Consequently, positive psychology interventions, which place high demands on the psychological expertise of implementers, may face challenges in being effectively carried out in schools in less developed regions. In the short term, establishing a mental health course specifically for implementing positive psychology interventions is impractical for some vocational secondary schools.

Given these challenges, embedding the PERMA model into traditional teaching practices may be a practical approach for its large-scale implementation PERMA model in vocational schools. In this study, the researchers adopted the widely applied PBL educational model in vocational high schools in mainland China as the instructional framework (Ding et al., 2014), guided by the PERMA model as the theoretical foundation. Using *Mechanical Drawing*, a compulsory course for mechanical majors in vocational high schools, as the curriculum content, a controlled experiment was conducted in a vocational high school in mainland China. The study aimed to develop a novel teaching method capable of facilitating the large-scale implementation of the PERMA model and positive psychology principles in vocational high school education.

## 2 Teaching framework

The PBL teaching model originated in the 1960s at McMaster University’s Faculty of Medicine in Canada. Over the course of half a century, its application has expanded beyond the field of medical education to various industries (Servant-Miklos et al., 2019). PBL emphasizes situating learning within real-world problem contexts, enabling learners to collaboratively solve problems and understand the underlying scientific principles and knowledge (Wood, 2003; Dolmans et al., 2005). The traditional PBL teaching model typically comprises five key steps: group division, ask questions, exploration and discussion, outcome presentation, and evaluation and reflection (Barrows and Tamblyn, 1977). Previous studies have shown that the PBL teaching model can significantly improve vocational key abilities, classroom satisfaction (Belwal et al., 2020), and creative thinking abilities (Aprilia et al., 2019) of vocational school students. It is an efficient teaching model that promotes students’ comprehensive development (Ren, 2022).

**Figure 1:**
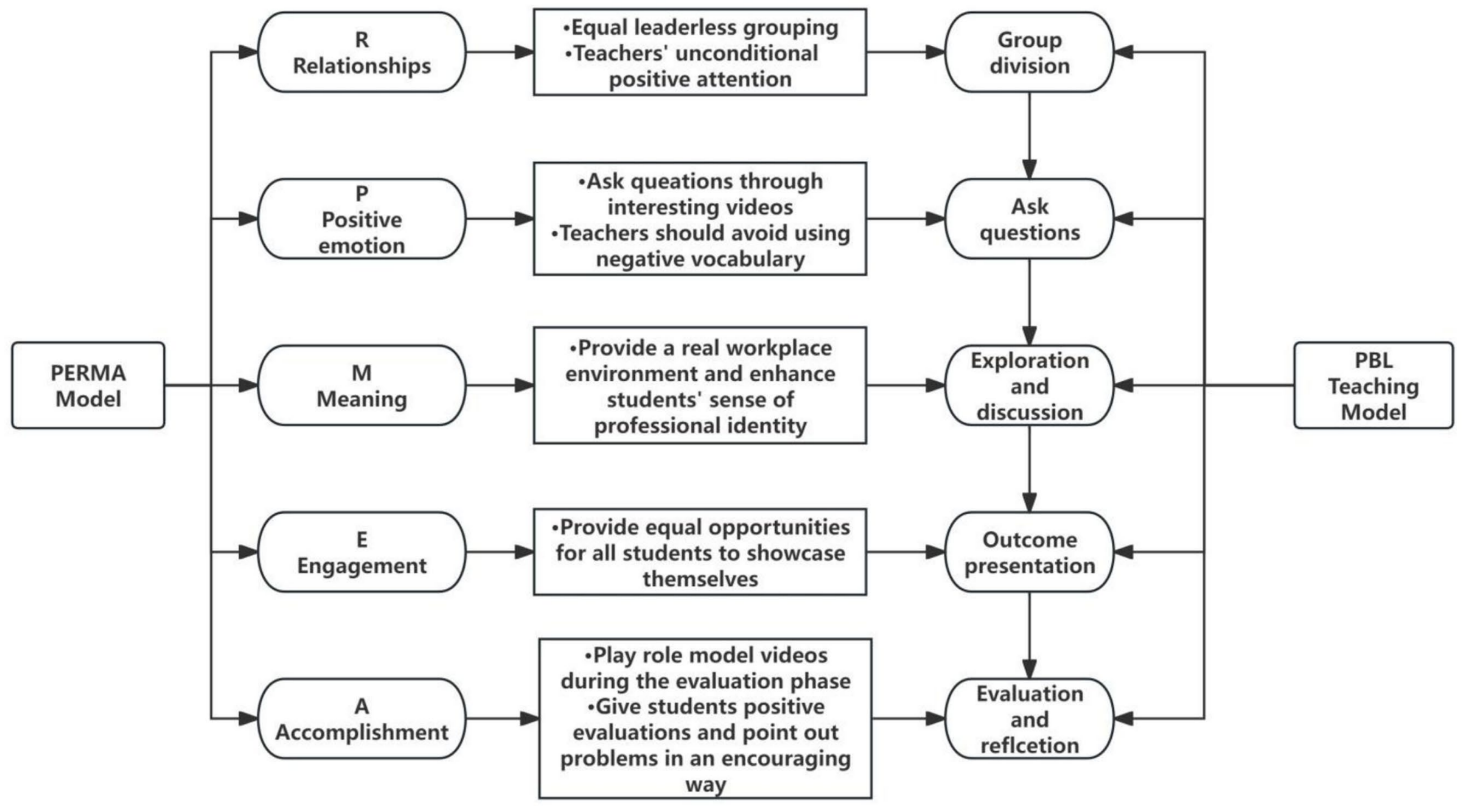
Teaching framework of the PERMA-based PBL teaching model.

The role of the PERMA model in enhancing students’ well-being and positive psychological qualities has been widely validated across different educational levels (Kovich et al., 2023; Kern et al., 2015). In this study, the five elements of the PERMA model were integrated into the five steps of the PBL teaching model to systematically optimize traditional PBL practices. The teaching framework is summarized in Figure 1.

The first key element of the PERMA model is Positive emotion (P). Positive emotion refers to experiences of good feelings such as gratitude, joy, and satisfaction (Seligman, 2012). It is regarded as a critical indicator of well-being (Coffey et al., 2015) and is directly associated with improvements in adolescent depression (Santos et al., 2013). Hence, improving vocational high school students’ positive experiences in class may enhance their mental health. A study has shown that watching amusing videos before learning significantly boosts learners’ positive emotions (Wang and Chen, 2022). Therefore, in the Ask Questions step of PBL, researchers showed students an engaging video, the video can be an animation or a movie clip to ensure that most students are interested. Additionally, during the entire teaching process, researchers avoided using negative evaluative language with all students.

The second key element of the PERMA model is Engagement (E), which emphasizes learners’ involvement in the learning process. High engagement contributes to improved academic performance and increased positive behaviors, making it a critical predictor of academic success (Ciric and Jovanovic, 2016). In traditional PBL, the Outcome Presentation phase usually completed by group representatives from each group. However, this practice may reduce the engagement of other group members. To address this, all group members in this study were required to present their respective contributions to the project in front of their peers and the teacher, ensuring equal attention and participation for all students.

The third key element is Relationships (R), focusing on love, recognition, and social support from others (Seligman, 2012). Positive interpersonal relationships during the learning process help students cope better with academic stress and achieve better outcomes (Davarniya et al., 2019; Kiuru et al., 2020). Based on this, group tasks were assigned in a democratic manner without designated leaders, ensuring that all group members only had distinct responsibilities without hierarchical roles. Positive teacher-student relationships can also significantly enhance student motivation (Zhang, 2022). Teacher enthusiasm plays a vital role in fostering such relationships. During group discussions, the researchers actively circulated among the groups, offering students encouragement and support, enabling students to feel the teacher’s warmth and energy.

The fourth key element is Meaning (M). Seligman defines meaning as the subjective sense of belonging to and serving something greater than oneself (Seligman, 2012). This element plays an important role in enhancing students’ academic performance and mental health (Mason, 2017; Hassed et al., 2009). Vocational identity strongly supports the sense of meaning, with higher vocational identity correlating with a stronger sense of belonging and purpose (Toubassi et al., 2023). During the Exploration and Discussion phase, researchers provided each group with sufficient models or tools derived from real workplace scenarios. In some instances, classes will be relocated to the school’s practical training center, allowing students to experience authentic work environments, thereby strengthening their vocational identity.

The fifth key element is Accomplishment (A). Accomplishment refers to the sense of achieving goals, which provides learners with motivation and self-efficacy, helping them maintain high levels of learning enthusiasm (Seligman, 2012; Lan and Saad, 2024). Research has shown that role models significantly enhance students’ sense of achievement, with a key source of self-efficacy and accomplishment being the observation of role models succeeding in similar tasks (Bandura, 2001; Gladstone and Cimpian, 2021). In the “Evaluation and Reflection” phase, researchers showed students a video showcasing exemplary figures in related fields to inspire them. Motivational teaching also enhances students’ sense of accomplishment and interest in learning (Cents-Boonstra et al., 2022). After group presentations, the researchers gave positive feedback on all student contributions. For well-executed parts, students were praised enthusiastically, while for weaker sections, they were encouraged with constructive suggestions on how to improve.

## 3 Materials and methods

### 3.1 Participants

The participants in this study were first-year vocational high school students from four parallel classes (Electromechanical Class 1, Electromechanical Class 2, Numerical Control Class 1, and Electromechanical 3 + 4 Class) of the 2023 cohort at a vocational secondary school in Shandong Province, China, totaling 241 students initially recruited. Given that all participants were from this locality, where primary and junior high schools exclusively utilize traditional teaching methods centered on lecture-based instruction, it can be reasonably assumed that none had prior exposure to the PERMA model or other related positive psychology models or theories. Furthermore, participation in this project was entirely voluntary, with no attendance requirements during the instructional sessions. Ultimately, 239 students from the four parallel classes voluntarily participated in the study. However, during the research process, 11 students failed to complete all assessments, and an additional 21 students submitted blank or invalid questionnaires for at least one assessment, ultimately yielding a final valid sample of 207 students.

### 3.2 Data collection and analysis tools

This study utilized the Inventory of Positive Mental Characters among Chinese Middle School Students developed by Meng et al. (2016). The scale comprises six subscales: Cognition (*KMO* = 0.738, *α* = 0.66), Emotion (*KMO* = 0.738, *α* = 0.76), Willpower (*KMO* = 0.731, *α* = 0.77), Self-discipline (*KMO* = 0.679, *α* = 0.64), Altruism (*KMO* = 0.763, *α* = 0.80), and Transcendence (*KMO* = 0.871, *α* = 0.86). It contains 63 items scored on a 5-point Likert scale ranging from “Not at all like me” to “Very much like me.” The scale measures positive psychological qualities across six dimensions, aligning well with the psychological characteristics of Chinese middle school students, and each subscale demonstrates sufficient reliability and validity.

Additionally, the researchers developed a questionnaire on the current state of teaching in the *Mechanical Drawing* course for vocational high schools. This was designed to facilitate a more detailed understanding and comparison of the learning conditions of participants in Groups A and B throughout the study. In 2021, the Shanghai Municipal Education Commission issued the *Implementation Methods for Comprehensive Quality Evaluation of Vocational High School Students in Shanghai*, which established a system comprising five modules and 22 indicators to evaluate the comprehensive quality of vocational students. This system reflects the characteristics of vocational education with high scientific and objective standards (Shanghai Municipal Education Commission, 2021). Given that *Mechanical Drawing* is only one specialized course within the training system for mechanical majors in vocational high schools, this study excluded three modules from the original evaluation system that were unrelated to this course. The remaining modules, “Skills and Academic literacy” and “Labor” and “Professional literacy,” were retained. Based on the core content of these two modules, four evaluation dimensions were extracted: “Learning interest,” “Learning attitude,” “Course comprehension,” and “Professional literacy.” Additionally, given the generally low learning motivation among vocation high school students and the significant impact of positive psychology on individual motivational states (Li, 2019; Pajares, 2001), this study incorporated the dimension of “Learning motivation” into the questionnaire. This was intended to examine the extent to which the PERMA-Based PBL Teaching Model enhances participants’ learning motivation. To gauge participants’ attitudes toward the innovative teaching model, the questionnaire added an “Attitude toward the new teaching model” dimension in the pre-test and an “Attitude toward the current teaching method” dimension in the mid-and post-tests. The final questionnaire comprised six dimensions: “Learning interest,” “Learning attitude,” “Course comprehension,” “Learning motivation,” “Attitude toward the new teaching model/current teaching method,” and “Professional literacy.” It included 17 items scored on a 5-point Likert scale ranging from “Strongly agree” to “Strongly disagree.” It is important to note that the questionnaire has two versions: a pre-test version and a combined mid-test and post-test version. The pre-test version included “Attitude toward the new teaching model” as the fifth dimension, while the combined mid-test and post-test version replaced it with “Attitude toward the current teaching method.” The other dimensions remained consistent across versions. Both versions demonstrated sufficient reliability (Pre-test: *KMO* = 0.862, *α* = 0.89; Mid-and Post-test: *KMO* = 0.877, *α* = 0.85).

For data analysis, SPSS Statistics 27.0 was used to analyze the data collected from both the scale and the questionnaire.

### 3.3 Grouping and research process

To strictly adhere to the principle of educational equity, the four parallel classes were divided into two groups, A and B, based on their initial test scores on the Inventory of Positive Mental Characters among Chinese Middle School Students (Meng et al., 2016), ensuring balanced levels of performance across the groups. Group A comprised Electromechanical Class 1 and Electromechanical Class 2, with a total of 104 students, while Group B included Numerical Control Class 1 and the Electromechanical 3 + 4 Class, with a total of 103 students.

**Figure 2:**
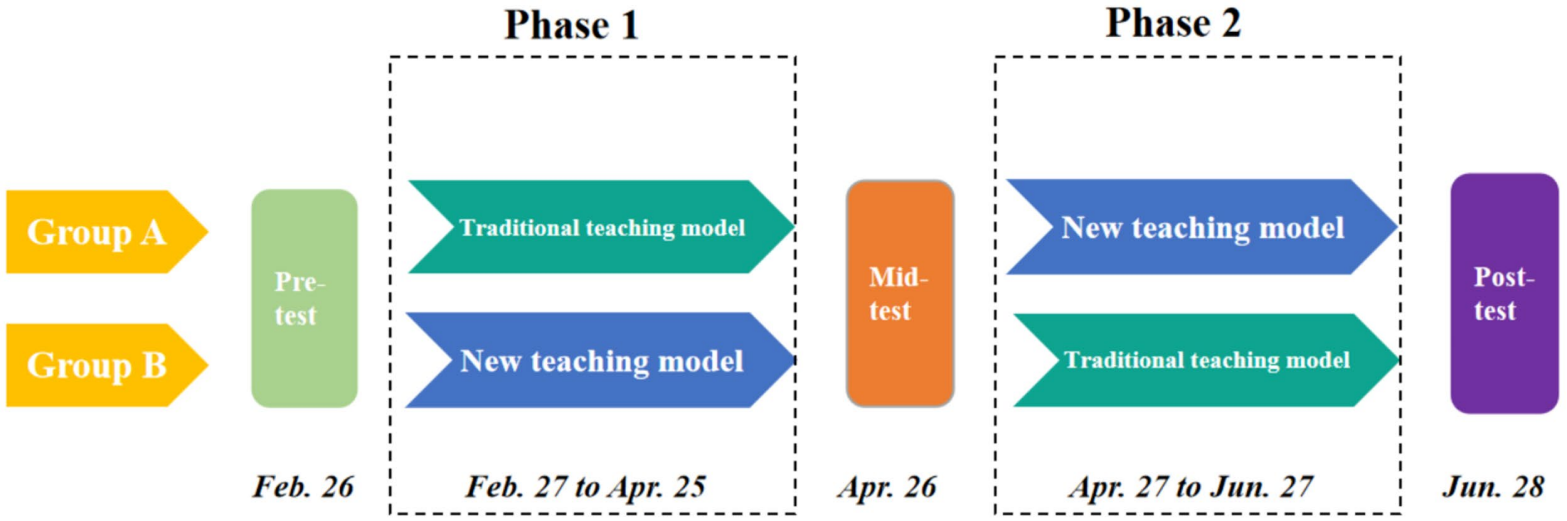
The research procedures for Group A and Group B.

As shown in Figure 2, the research employed a crossover experimental design divided into two phases. The study commenced on February 26, 2024, and concluded on June 28, 2024. The first phase spanned from February 27 to April 25, and the second phase from April 27 to June 27. The pre-test, mid-test, and post-test were administered on February 26, April 26, and June 28, respectively. During the research process, Groups A and B commenced the first phase the day after completing the pre-test. In this first phase, Group A followed the traditional teaching model, while Group B implemented the PERMA-based PBL teaching model. The mid-test was conducted the day after the conclusion of the first phase. Following the mid-test, Groups A and B commenced the second phase the next day. During this second phase, Group A transitioned to the new teaching model, while Group B switched to the traditional teaching model. The post-test was administered the day after the conclusion of the second phase.

## 4 Results

### 4.1 Results from the inventory of positive mental characters among Chinese middle school students

Table 1 presents the changes in positive psychological qualities of participants in Groups A and B throughout the experimental process. In the first phase of the study, the traditional teaching model used by Group A had a moderately significant effect (*p* < 0.05) on improving participants’ positive psychological qualities only in the “Cognition” dimension, with no significant effects observed in the other dimensions. In contrast, the PERMA-based PBL teaching model used by Group B highly significant improved (*p* < 0.001) all six dimensions of positive psychological qualities measured by the scale.

**Table 1:** Paired sample *t*-test data for positive psychological qualities within Groups A and B.

	Dimensions	Pre-test		Mid-test			Post-test		
		*M*	*SD*	*M*	*SD*	*t*	*M*	*SD*	*t*
Group A *N* = 204	Cognition	2.98	0.99	3.09	1.00	2.50*	3.53	0.99	10.15***
	Emotion	3.24	1.06	3.26	1.09	0.45	3.74	0.99	10.54***
	Willpower	3.01	0.98	3.08	1.01	1.66	3.53	1.02	10.59***
	Self-discipline	3.21	1.05	3.22	1.06	0.18	3.61	0.98	8.52***
	Altruism	3.12	1.06	3.16	1.02	0.92	3.61	0.95	10.26***
	Transcendence	3.26	1.09	3.29	1.03	0.77	3.77	0.96	12.75***
Group B *N* = 203	Cognition	2.97	1.02	3.45	0.95	11.33***	3.54	0.87	2.07*
	Emotion	3.24	1.14	3.66	1.00	8.95***	3.74	0.88	2.04*
	Willpower	3.01	1.07	3.45	0.99	10.26***	3.56	0.87	3.02**
	Self-discipline	3.20	1.14	3.54	0.98	7.58***	3.62	0.90	2.12*
	Altruism	3.14	1.12	3.58	0.97	9.27***	3.62	0.89	1.06
	Transcendence	3.27	1.15	3.68	0.92	10.35***	3.77	0.84	2.61**

*: *p* < 0.05; **: *p* < 0.01; ***: *p* < 0.001.

During the second phase, Group A, which transitioned to the new teaching model, exhibited highly significant improvements across all six dimensions of positive psychological qualities. Meanwhile, Group B, which switched to the traditional teaching model, showed moderately significant improvements in the “Cognition,” “Emotion” and “Self-discipline” dimensions, and significant improvements (*p* < 0.01) in the “Willpower” and “Transcendence” dimensions.

Table 2 presents the intergroup differences in the positive psychological qualities of participants in Groups A and B during the experiment. Since the four parallel classes were grouped using a balanced approach based on their initial scores, and neither group had prior exposure to positive psychology theories, the pre-test scores of Groups A and B were nearly identical. At the end of the first phase, the positive psychological qualities of Group B participants, who received the new teaching model, were highly significantly higher across all six dimensions compared to those of Group A participants, who followed the traditional teaching model. By the end of the second phase, the positive psychological qualities of Groups A and B converged, with no significant differences observed between the two groups.

**Table 2:** Independent sample *t*-test data for positive psychological qualities between Groups A and B.

Dimensions	Pre-test			Mid-test			Post-test		
	*M_A_*	*M_B_*	*t*	*M_A_*	*M_B_*	*t*	*M_A_*	*M_B_*	*t*
Cognition	2.98	2.97	0.20	3.09	3.46	−8.57***	3.53	3.54	−0.20
Emotion	3.24	3.24	0.01	3.26	3.66	−8.67***	3.74	3.74	−0.06
Willpower	3.01	3.01	−0.11	3.08	3.45	−8.83***	3.53	3.56	−0.75
Self-discipline	3.21	3.20	0.26	3.22	3.54	−7.16***	3.61	3.62	−0.40
Altruism	3.12	3.14	−0.32	3.16	3.58	−8.94***	3.61	3.62	−0.316
Transcendence	3.26	3.27	−0.120	3.29	3.68	−10.38***	3.77	3.77	0.06

*: *p* < 0.05; **: *p* < 0.01; ***: *p* < 0.001.

### 4.2 Results from the course survey questionnaire

Table 3 presents the changes in the learning states of participants in Groups A and B throughout the experiment. In the first phase of the study, the traditional teaching model employed in Group A moderately significantly enhanced participants’ “Professional literacy,” while improvements in other dimensions were not statistically significant. In contrast, the novel teaching model applied in Group B produced highly significant improvements in participants’ “Learning interest” and “Learning motivation,” significant improvements in their “Course comprehension,” and moderately significant enhancements in their “Learning attitude.” However, it did not lead to significant improvements in participants’ “Professional literacy.”

**Table 3:** Paired sample *t*-test data for teaching status within Groups A and B.

	Dimensions	Pre-test		Mid-test			Post-test		
		*M*	*SD*	*M*	*SD*	*t*	*M*	*SD*	*t*
Group A *N* = 204	Learning interest	3.14	0.95	3.28	0.83	1.84	3.76	0.96	6.63***
	Learning attitude	3.20	0.99	3.32	0.91	1.26	3.69	0.90	4.40***
	Course comprehension	3.43	1.00	3.54	0.82	1.20	3.82	0.89	3.35***
	Learning motivation	2.81	0.88	2.95	0.81	1.26	3.54	0.93	9.75***
	Attitude toward the new teaching model	3.31	0.97	/	/	/	/	/	/
	Attitude toward the current teaching method	/	/	3.30	1.01	/	3.76	0.91	7.30***
	Professional literacy	3.58	0.91	3.74	0.79	1.99*	4.04	0.82	3.84***
Group B *N* = 203	Learning interest	3.10	0.92	3.61	0.88	7.18***	3.73	0.82	1.71
	Learning attitude	3.32	1.02	3.53	0.82	2.37*	3.69	0.80	1.90
	Course comprehension	3.33	0.98	3.60	0.90	2.97**	3.80	0.78	2.37*
	Learning motivation	2.91	0.94	3.37	0.96	7.19***	3.57	1.05	2.86**
	Attitude toward the new teaching model	3.38	0.98	/	/	/	/	/	/
	Attitude toward the current teaching method	/	/	3.80	0.85	/	3.22	1.02	−8.42***
	Professional literacy	3.62	0.95	3.78	0.81	1.89	4.08	0.73	3.90***

*: *p* < 0.05; **: *p* < 0.01; ***: *p* < 0.001.

In the second phase, participants in Group A exhibited highly significant improvements across all six dimensions of learning states measured by the questionnaire. For Group B, compared to their mid-test levels, participants showed highly significant improvement in “Professional literacy,” significant improvement in “Learning motivation,” moderately significant improvement in “Course comprehension,” but a highly significant decline in their “Attitude toward the current teaching method.” Additionally, in the pre-test, the scores for “Attitude toward the new teaching model” were 3.31 and 3.38 for Groups A and B, respectively, exceeding the theoretical midpoint by 10.3 and 12.7%.

Table 4 presents the intergroup differences in the learning states of participants in Groups A and B during the experiment. As shown, there were no significant differences between Groups A and B in their learning states during the pre-test. After the first phase of teaching, Group B participants scored significantly higher than Group A in three dimensions: “Learning interest,” “Learning motivation,” and “Attitude toward the current teaching method,” with highly significant differences. In the “Learning attitude” dimension, Group B also scored moderately significantly higher than Group A. After the second phase, Group A participants exhibited a highly significant advantage over Group B in their “Attitude toward the current teaching method.” Beyond this, no significant differences were observed between the two groups.

**Table 4:** Independent sample *t*-test data for teaching status between Groups A and B.

Dimensions	Pre-test			Mid-test			Post-test		
	*M_A_*	*M_B_*	*t*	*M_A_*	*M_B_*	*t*	*M_A_*	*M_B_*	*t*
Learning interest	3.14	3.10	0.63	3.28	3.61	−4.84***	3.76	3.73	0.49
Learning attitude	3.20	3.32	−1.20	3.32	3.53	−2.49*	3.69	3.69	0.03
Course comprehension	3.43	3.33	1.05	3.54	3.60	−0.64	3.82	3.80	0.26
Learning motivation	2.81	2.91	−1.55	2.95	3.37	−6.86***	3.54	3.57	−0.47
Attitude toward the new teaching model	3.31	3.38	−1.08	/	/	/	/	/	/
Attitude toward the current teaching method	/	/		3.30	3.80	−7.77***	3.76	3.22	8.16***
Professional literacy	3.58	3.62	−0.43	3.74	3.78	−0.52	4.04	4.08	−0.45

*: *p* < 0.05; **: *p* < 0.01; ***: *p* < 0.001.

## 5 Discussion

### 5.1 The impact of the PERMA-based PBL teaching model on students’ positive psychological qualities

It is evident that the PERMA-based PBL teaching model outperformed the traditional teaching model in enhancing the positive psychological qualities of vocational high school students, aligning with findings from studies across other educational levels (Kovich et al., 2023; Kern et al., 2015). Instruction guided by the PERMA model provides students with increased positive emotional experiences and overall well-being, thereby fostering individual and societal flourishing (Dorri and Aghaei, 2024).

However, as shown in Table 1, the efficacy of the traditional teaching model in improving participants’ positive psychological qualities differed between Groups A and B. In Group A, the traditional teaching model implemented during the first phase only yielded moderately significant improvements in participants’ “Cognitive” levels. Conversely, in Group B, the traditional teaching model used during the second phase resulted in statistically significant enhancements across five dimensions: “Cognition,” “Emotion,” “Willpower,” “Self-discipline,” and “Transcendence.” This discrepancy may be attributed to the new teaching model applied to Group B during the first phase, which established more positive teacher-student relationships prior to their exposure to traditional instruction. Consequently, even without incorporating positive psychology theories in subsequent teaching, researchers still exerted a beneficial influence on this group’s positive psychological qualities. In classroom settings, negative teacher-student relationships undermine students’ autonomy and subjective well-being, ultimately impairing their academic performance and mental health (Zhou et al., 2023). Conversely, positive teacher-student relationships significantly boost students’ well-being, learning motivation, and academic achievement—a finding consistent with this study’s experimental results (Robinson, 2022).

Collectively, the PERMA-based PBL teaching model not only provides vocational students with more positive emotional experiences but also facilitates stronger teacher-student relationships, thus comprehensively enhancing their positive psychological qualities.

### 5.2 The impact of the PERMA-based PBL teaching model on students’ learning states

Based on the data presented in Tables 3, 4, it is evident that vocational high school students demonstrate a stronger preference for the novel teaching model. Furthermore, the novel teaching model exhibits significantly greater effectiveness than the traditional teaching model in enhancing students’ learning interest and learning attitudes, thereby reconfirming the role of positive psychology theory in improving students’ learning states (Al-Mansoori and Dana, 2017).

However, as illustrated in Table 4, the advantages of the novel teaching model over the traditional model in improving students’ “Course comprehension” and “Professional literacy” are less pronounced. Regarding “Course comprehension,” the novel teaching model highly significantly enhanced this dimension among participants in Group A and significantly enhanced it in Group B. In contrast, the traditional teaching model had a moderately significant effect on improving course comprehension in Group B but showed no significant effect in Group A. This indicates that the novel teaching model outperforms the traditional model in enhancing participants’ comprehension of the course. One reason for the non-significant results in the independent-samples t-test of the mid-test mean scores for the “Course comprehension” dimension between Groups A and B is that the pre-test mean score for this dimension in Group B was lower than that in Group A.

Concerning “Professional literacy,” Table 3 reveals minimal improvement in this dimension for both groups during the first phase of the study, whereas highly significant enhancement was observed in the second phase. This pattern may be attributed to the logical structure of the Mechanical Drawing course content. In mainland China, this course is typically taught in a sequence of theory followed by practice (Zhu and Xu, 2020). Consequently, the teaching content in the second phase incorporated substantially more practice-oriented tasks closely aligned with real workplace environments compared to the first phase. Since practice serves as an effective means to enhance engineering students’ innovation capabilities and professional literacy (Chen et al., 2021), this discrepancy may have led to an uneven rate of improvement in students’ professional literacy levels between the two phases. Additionally, participants in this study were simultaneously enrolled in other specialized courses during the research period, which may have also influenced their professional literacy levels.

As technology continues to advance, society demands increasingly higher levels of professional literacy from vocational high school students. How to comprehensively enhance students’ professional literacy during the teaching process and strengthen their competitiveness in the job market remains a significant educational challenge (Huang et al., 2011).

### 5.3 Limitations

The participants in this study were exclusively first-year mechanical students from a vocational high school in Shandong Province, with no inclusion of students from other disciplines or regions. This limits the generalizability of the findings to a broader student population. Furthermore, in mainland China, male students significantly outnumber females in vocational high school mechanical programs. As a result, this study did not specifically explore gender as a factor.

### 5.4 Recommendations

This study confirmed the effectiveness of the PERMA-based PBL teaching model. Future research should aim to extend the application of this teaching model to other disciplines, educational stages, and countries or regions while exploring demographic variables in greater detail to evaluate and refine the model further.

Additionally, leveraging positive psychology theories or models as guiding frameworks to reform traditional teaching practices represents a promising direction for positive education. Future studies could adaptively select various positive psychology theories and compatible teaching models based on course characteristics to achieve optimal teaching outcomes.

Finally, as an important framework within positive psychology, the PERMA model is undoubtedly effective in improving individual health and well-being. However, its five core elements are not absolutely comprehensive and may benefit from refinement. Future researchers could consider modifying the dimensions of the PERMA model to develop a more holistic approach to enhancing participants’ positive psychological qualities.

## 6 Conclusion

This study proposed a PERMA-based PBL teaching model by integrating the PERMA framework into the PBL teaching approach. Compared to traditional positive psychology interventions, which often require high levels of psychological expertise from implementers, this teaching model is relatively simple to operate, demands less knowledge of positive psychology theories, and is more feasible for widespread implementation in vocational high schools.

Using a crossover experimental design, this study validated the effectiveness of the new teaching model. The PERMA-based PBL teaching model significantly outperformed the traditional teaching model across all six dimensions of positive psychological qualities. Additionally, it proved more effective than the traditional teaching model in enhancing students’ “Learning Interest,” “Learning Attitude,” and “Learning Motivation.” Overall, the novel teaching model significantly enhances the positive psychological qualities of vocational high school students and improves their overall learning conditions. Moreover, compared to the traditional teaching model, the novel teaching model is more favorably received among vocational high school students.

## Data Availability

The original contributions presented in the study are included in the article/supplementary material, further inquiries can be directed to the corresponding author.

## References

[ref1] Al-Mansoori F. K. F. Dana H. (2017). Abdeen implementing positive education in a preparatory school: a case study from Qatar. Int. J. Inf. Educ. Technol. 7, 654–660.

[ref2] Aprilia T. Widaningsih L. Megayanti T.. (2019). Implementation of learning models of problem-based learning to improve the creative thinking ability of vocational students, In Proceedings of the 5th UPI International Conference on Technical and Vocational Education and Training (ICTVET 2018), 269–272

[ref3] Bandura A. (2001). Social cognitive theory: an agentic perspective. Annu. Rev. Psychol. 52, 1–26. doi: 10.1146/annurev.psych.52.1.1, PMID: 11148297

[ref4] Barrows H. S. Tamblyn R. M. (1977). The portable patient problem pack: a problem-based learning unit. J. Med. Educ. 52, 1002–1004. doi: 10.1097/00001888-197712000-00020 926146

[ref1000] Belwal R. Belwal S. Sufian A. B. Al Badi A. (2020). Project-based learning (PBL): outcomes of students’ engagement in an external consultancy project in Oman. Educ. Train. 63, 336–359. doi: 10.1108/ET-01-2020-0006

[ref5] Cents-Boonstra M. Lichtwarck-Aschoff A. Lara M. M. Denessen E. (2022). Patterns of motivating teaching behaviour and student engagement: a microanalytic approach. Eur. J. Psychol. Educ. 37, 227–255. doi: 10.1007/s10212-021-00543-3

[ref6] Chen W. P. Lin Y. X. Ren Z. Y. Shen D. (2021). Exploration and practical research on teaching reforms of engineering practice center based on 3I-CDIO-OBE talent-training mode. Comput. Appl. Eng. Educ. 29, 114–129. doi: 10.1002/cae.22248

[ref7] Chim K. Lai J. T. C. Chan B. T. Y. (2024). Embedding positive psychology into curriculum to promote posttraumatic growth, psychological flexibility, and socio-emotional competencies in higher education. Front. Psychol. 15:1450192. doi: 10.3389/fpsyg.2024.1450192, PMID: 39399264 PMC11466883

[ref8] Chue K. L. Yeo A. Nie Y. Chew L. C. (2024). Modifying the PERMA profiler to assess student well-being. Curr. Psychol. 43, 3749–3760. doi: 10.1007/s12144-023-04550-z

[ref9] Ciric M. Jovanovic D. (2016). Student engagement as a multidimensional concept. Logos Universality Ment. Educ. Nov 15, 187–194. doi: 10.15405/epsbs.2016.11.20

[ref10] Coffey J. K. Warren M. T. Gottfried A. W. (2015). Does infant happiness forecast adult life satisfaction? Examining subjective well-being in the first quarter century of life. J. Happiness Stud. 16, 1401–1421. doi: 10.1007/s10902-014-9556-x

[ref11] Davarniya R. Shakarami M. Zahrakar K. (2019). Resilience, coping strategies, and social support: important predictors of students’ vulnerability to stress. J. Res. Health. 9, 90–94. doi: 10.32598/JRH.9.1.90

[ref9004] Ding X. Zhao L. Chu H. Tong N. Ni C. Hu Z. . (2014). Assessing the Effectiveness of Problem-Based Learning of Preventive Medicine Education in China. Sci Rep. 4:5126. doi: 10.1038/srep05126 24874915 PMC4038805

[ref12] Dolmans D. H. J. M. De Grave W. Wolfhagen I. H. A. P. van der Vleuten C. P. M. (2005). Problem-based learning: future challenges for educational practice and research. Med. Educ. 39, 732–741. doi: 10.1111/j.1365-2929.2005.02205.x, PMID: 15960794

[ref13] Dong L. Peng L. Wang Y. (2020). The current status of professional quality development of rural primary school teachers: an investigation in Hebei Province. J. Tianjin Acad. Educ. Sci. 3, 82–88.

[ref14] Dorri S. S. Aghaei A. (2024). The effectiveness of PERMA model education on university students’ well-being. J. Educ. Health Promot. 13:338. doi: 10.4103/jehp.jehp_840_23 39679036 PMC11639425

[ref15] Falecki D. Leach C. Green S. (2018). “PERMA-powered coaching: Building foundations for a flourishing life” in Positive psychology coaching in practice. eds. Green S. Palmer S. (London: Routledge Press), 23–39.

[ref16] Gladstone J. R. Cimpian A. (2021). Which role models are effective for which students? A systematic review and four recommendations for maximizing the effectiveness of role models in STEM. Int. J. STEM Educ. 8:59. doi: 10.1186/s40594-021-00315-x 34868806 PMC8636406

[ref17] Global Happiness Council. (2018). Global happiness policy report 2018. Available online at: https://www.happinesscouncil.org/report/2018/global-happiness-policy-report (Accessed May 23, 2024).

[ref18] Hassed C. De Lisle S. Sullivan G. Pier C. (2009). Enhancing the health of medical students: outcomes of an integrated mindfulness and lifestyle program. Adv. Health Sci. Educ. 14, 387–398. doi: 10.1007/s10459-008-9125-3, PMID: 18516694

[ref19] He Y. Zeng Q. Zhang M. (2023). The mediating roles of future work self and Hope on the association between perceived social support and depressive symptoms among Chinese vocational school students: a cross-sectional study. Psychol. Res. Behav. Manag. 16, 2125–2136. doi: 10.2147/PRBM.S414356, PMID: 37334406 PMC10275316

[ref20] Horváth L. O. Balint M. Ferenczi-Dallos G. Farkas L. Gadoros J. Gyori D. . (2018). Direct self-injurious behavior (D-SIB) and life events among vocational school and high school students. Int. J. Environ. Res. Public Health 15:1068. doi: 10.3390/ijerph15061068, PMID: 29795028 PMC6025121

[ref22] Huang X. X. Wang L. T. Xu H. X. (2011). A study on vocational computer science students’ professionalism culture. Adv. Mater. Res. 403-408, 2890–2892. doi: 10.4028/www.scientific.net/amr.403-408.2890

[ref23] Kern M. L. Waters L. E. Adler A. White M. A. (2015). A multidimensional approach to measuring well-being in students: application of the PERMA framework. J. Posit. Psychol. 10, 262–271. doi: 10.1080/17439760.2014.936962, PMID: 25745508 PMC4337659

[ref24] King R. B. Caleon I. S. Bernardo A. B. I. (2023). Positive psychology and positive education: Asian perspectives on well-being in schools. Singapore: Springer Press.

[ref25] Kiuru N. Wang M. T. Salmela-Aro K. Kannas L. Ahonen T. Hirvonrn R. (2020). Associations between adolescents’ interpersonal relationships, school well-being, and academic achievement during educational transitions. J. Youth Adolesc. 49, 1057–1072. doi: 10.1007/s10964-019-01184-y 31893326 PMC7182546

[ref26] Kovich M. K. Simpson V. L. Foli K. J. Hass Z. Phillips R. G. (2023). Application of the PERMA model of well-being in undergraduate students. Int. J. Community Well-Being 6, 1–20. doi: 10.1007/s42413-022-00184-4, PMID: 36320595 PMC9607835

[ref27] Lai M. K. Leung C. Kwok S. Y. C. Hui A. N. N. Lo H. H. M. Leung J. T. Y. . (2018). A multidimensional PERMA-H positive education model, general satisfaction of school life, and character strengths use in Hong Kong senior primary school students: confirmatory factor analysis and path analysis using the APASO-II. Front. Psychol. 9:1090. doi: 10.3389/fpsyg.2018.01090, PMID: 30008690 PMC6034423

[ref28] Lan Y. Saad M. R. B. (2024). Emotional presence in the community of inquiry: addition of PERMA in online English teaching and learning. Asia-Pac. Educ. Res. 34, 732–737. doi: 10.1007/s40299-024-00891-w

[ref29] Leow T. Li W. W. Miller D. J. McDermott B. (2024). Prevalence of university non-continuation and mental health conditions, and the effect of mental health conditions on non-continuation: a systematic review and meta-analysis. J. Ment. Health. 34, 222–237. doi: 10.1080/09638237.2024.2332812 38588717

[ref30] Li D. (2019). Investigation report on the psychological learning problems of post-2000 vocational students. China Educ. Technol. Equip. 19, 31–33.

[ref9005] Li F. Cui Y. Li Y. Guo L. Ke X. Liu J. . (2022). Prevalence of mental disorders in school children and adolescents in China: diagnostic data from detailed clinical assessments of 17,524 individuals. J Child Psychol Psychiatr. 63, 34–46. doi: 10.1111/jcpp.13445 34019305

[ref31] Lim E. W. C. (2024). “Educators’ perspectives in implementing positive education,” in Enhancing holistic well-being of children and youth, ed. Tan O. S. Low E. L. Caleon I. S. Ng E. L. (Singapore, Springer Press), 237–250

[ref33] Liu S. Jiang Z. (2020). Stimulating and maintaining learning motivation in vocational students: a comparative study of vocational and high school students' learning motivation. Sci. Educ. Cult. (Mid-Mon. Ed.). 8, 159–161.

[ref1001] Lu J. Tan M. Ma Y. Li J. Yang F. Zhou X. (2025). Better mental health and better academic performance: A longitudinal study of high school freshmen in China. J. Affect. Disord. 386:119424. doi: 10.1016/j.jad.2025.119424 40383304

[ref34] Lyubomirsky S. King L. Diener E. (2005). The benefits of frequent positive affect: does happiness lead to success? Psychol. Bull. 131, 803–855. doi: 10.1037/0033-2909.131.6.803, PMID: 16351326

[ref35] Mason H. D. (2017). Sense of meaning and academic performance: a brief report. J. Psychol. Afr. 27, 282–285. doi: 10.1080/14330237.2017.1321860

[ref36] Meng W. Zhang C. Wagner R. (2016). Revision report of the positive psychological quality scale for Chinese middle school students. Chin. J. Spec. Educ. 2, 69–73+79.

[ref37] Morgan B. Simmons L. (2021). A "PERMA" response to the pandemic: an online positive education programme to promote wellbeing in university students. Front. Educ. 6:642632. doi: 10.3389/feduc.2021.642632

[ref38] Pajares F. (2001). Toward a positive psychology of academic motivation. J. Educ. Res. 95, 27–35. doi: 10.1080/00220670109598780

[ref40] Ren Y. (2022). Knowledge spillover and emotional motivation: a study on the willingness and influencing factors of project-based learning. Front. Psychol. 13:795552. doi: 10.3389/fpsyg.2022.795552, PMID: 35664171 PMC9157245

[ref41] Robinson C. D. (2022). A framework for motivating teacher-student relationships. Educ. Psychol. Rev. 34, 2061–2094. doi: 10.1007/s10648-022-09706-0

[ref42] Santos V. Paes F. Pereira V. Arias-Carrion O. Silva A. C. Carta M. G. . (2013). The role of positive emotion and contributions of positive psychology in depression treatment: systematic review. Clin. Pract. Epidemiol. Ment. Health 9, 221–237. doi: 10.2174/1745017901309010221, PMID: 24358052 PMC3866689

[ref43] Seligman M. E. (2012). A visionary new understanding of happiness and well-being. New York, NY: Simon & Schuster Press.

[ref44] Servant-Miklos V. F. C. Woods N. N. Dolmans D. H. J. M. (2019). Celebrating 50 years of problem-based learning: progress, pitfalls, and possibilities. Adv. Health Sci. Educ. 24, 849–851. doi: 10.1186/s12889-024-20271-9 31832932

[ref2000] Shanghai Municipal Education Commission. (2021). Notice of the Shanghai Municipal Education Commission on Issuing the Implementation Measures for the Comprehensive Quality Evaluation of Students in Secondary Vocational Schools in Shanghai. https://edu.sh.gov.cn/xxgk2_zdgz_rxgkyzs/20230420/9768290ae3b04666a1522e32bd45b732.html [Accessed June 30, 2025]

[ref45] Su Y. Chen Z. Teng Z. Liu M. D. Yang Y. G. Chen J. D. . (2024). The relationship between childhood trauma and mental health status among Chinese vocational high school adolescents: the mediating effect of poor self-control and internet addiction. BMC Public Health 24:2747. 10.1186/s12889-024-20271-9 PMC11462986 39379860

[ref47] Toubassi D. Schenker C. Roberts Forte M. (2023). Professional identity formation: linking meaning to well-being. Adv. Health Sci. Educ. 28, 305–318. doi: 10.1007/s10459-022-10146-2 PMC9341156 35913664

[ref48] Wang M. Chen Z. (2022). Laugh before you study: does watching funny videos before study facilitate learning? Int. J. Environ. Res. Public Health 19:4434. doi: 10.3390/ijerph19084434, PMID: 35457302 PMC9030648

[ref49] Wood D. F. (2003). Problem-based learning. BMJ 326, 328–330. doi: 10.1136/bmj.326.7384.328, PMID: 12574050 PMC1125189

[ref50] World Health Organization. (2022). World mental health report: transforming mental health for all. Available online at: https://www.who.int/publications/i/item/9789240049338 (Accessed May 30, 2024)

[ref54] Zhang Q. (2022). The role of teachers’ interpersonal behaviors in learners’ academic achievements. Front. Psychol. 13:921832. doi: 10.3389/fpsyg.2022.921832, PMID: 35783768 PMC9247453

[ref56] Zhao Y. Yu F. Wu Y. Zeng G. Peng K. (2019). Positive education interventions prevent depression in Chinese adolescents. Front. Psychol. 10:1344. doi: 10.3389/fpsyg.2019.01344, PMID: 31249543 PMC6582777

[ref57] Zhou D. Liu S. Zhou H. Liu J. Ma Y. (2023). The association among teacher-student relationship, subjective well-being, and academic achievement: evidence from Chinese fourth graders and eighth graders. Front. Psychol. 14:1097094. doi: 10.3389/fpsyg.2023.1097094, PMID: 36777196 PMC9909438

[ref59] Zhu Q. Xu G. (2020). A comparative study on the compilation of mechanical vocational education textbooks between China and Japan: taking vocational “mechanical drawing” textbooks as an example. Vocat. Educ. Forum. 36, 78–86.

